# Viral Proteins Involved in the Adsorption Process of Deep-Purple, a Siphovirus Infecting Members of the Bacillus cereus Group

**DOI:** 10.1128/aem.02478-21

**Published:** 2022-05-02

**Authors:** Audrey Leprince, Manon Nuytten, Jacques Mahillon

**Affiliations:** a Laboratory of Food and Environmental Microbiology, Earth and Life Institute, Université catholique de Louvain, Louvain-la-Neuve, Belgium; Unversidad de los Andes

**Keywords:** adsorption, distal tail protein, phages, receptor binding protein, *Siphoviridae*

## Abstract

The infection of a bacterium by a tailed phage starts from the adsorption process, which consists of a specific and strong interaction between viral proteins called receptor binding proteins (RBPs) and receptors located on the bacterial surface. In addition to RBPs, other tail proteins, such as evolved distal tail (evoDit) proteins and tail lysins, harboring carbohydrate binding modules (CBMs) have been shown to facilitate the phage adsorption by interacting with host polysaccharides. In this work, the proteins involved in the adsorption of Deep-Purple, a siphovirus targeting bacteria of the Bacillus cereus group, were studied. Bioinformatic analysis of Deep-Purple tail protein region revealed that it contains two proteins presenting CBM domains: Gp28, an evoDit protein, and Gp29, the potential RBP. The implication of both proteins in the adsorption of Deep-Purple particles was confirmed through cell wall decoration assays. Interestingly, whereas RBP-Gp29 exhibited the same host spectrum as Deep-Purple, evoDit-Gp28 was able to bind to many B. cereus group strains, including some that are not sensitive to the phage infection. Using immunogold microscopy, both proteins were shown to be located in the phage baseplate. Additionally, an *in silico* analysis of the tail regions encoded by several *Siphoviridae* infecting the B. cereus group was performed. It revealed that although the tail organization displayed by Deep-Purple is the most prevalent, different tail arrangements are observed, suggesting that distinct baseplate organization and adsorption mechanisms are encountered in siphoviruses targeting the B. cereus group.

**IMPORTANCE** The B. cereus group is a complex cluster of closely related species, among which certain strains can be pathogenic (i.e., Bacillus anthracis, Bacillus cereus *sensu stricto*, and Bacillus cytotoxicus). Nowadays, phages are receiving increasing attention for applications in controlling and detecting such pathogens. Thus, understanding the molecular mechanisms governing the phage adsorption to its bacterial host is paramount as this step is a key determinant of the phage host spectrum. Until now, the knowledge regarding the adsorption process of tailed phage targeting the B. cereus groups was mainly restricted to the phage gamma infecting B. anthracis. With this work, we provide novel insights into the adsorption of Deep-Purple, a siphovirus infecting the B. cereus group. We showed that this phage recognizes polysaccharides and relies on two different viral proteins for its successful adsorption.

## INTRODUCTION

The infective cycle of virulent phages is initiated by the specific interaction between viral components, called receptor binding proteins (RBPs), and bacterial receptors on the cell surface, a process commonly depicted as the adsorption step (1). As a key determinant of the host spectrum and subsequent intracellular replication of the virus, this stage is one of the primary targets of resistance mechanisms developed by bacteria to overcome phage infection. For instance, they can alter or mask the receptor or even produce competitive inhibitors to prevent adsorption (2). It is therefore essential to better understand the molecular mechanisms governing phages’ adsorption to their bacterial hosts.

The *Caudovirales* order gathers double-stranded DNA (dsDNA) phages possessing a tail involved in the recognition of and adsorption to bacteria (3). More specifically, the tail end forms a structure called the baseplate on which RBPs (i.e., fiber, spike, and baseplate proteins) are anchored, together with proteins with peptidoglycan (PG)-degrading and depolymerase activities that facilitate adsorption and crossing of the cell envelope barriers (4–7). While RBPs targeting Gram-negative bacteria have been characterized in great detail (3), those associated with Gram-positive phages remain far less studied. Research has mainly focused on phages infecting Listeria monocytogenes, Staphylococcus aureus, Bacillus subtilis, and lactic acid bacteria (LAB), the latter being responsible for important economic losses in the dairy industry (8–12).

The best-characterized Gram-positive phage RBPs are those of siphoviruses (harboring a long noncontractile tail) infecting Lactococcus lactis, for which the structure of the adsorption apparatus and nature of the receptor are now available (13–15). Typically, their baseplate is formed by a distal tail (Dit) protein, delimiting a central hexameric structure onto which the tail tube and proteins are assembled. These baseplate proteins involve not only the RBP but also the tail-associated lysin (Tal), which is responsible for the local degradation of PG. In some cases, other structural components such as the upper and accessory baseplate proteins (13, 16) are also present. L. lactis phages are highly specific and mostly recognize surface-associated polysaccharides forming a protective layer called the pellicle (15, 17). Interestingly, these phages can be grouped based on the type of polysaccharides they recognize (18).

Recently, “evolved Dit” (evoDit) proteins have also been identified in lactococcal phages and appear to be implicated in host recognition (10). These particular Dit proteins possess a domain of about 150 amino acids (aa) consisting of a carbohydrate binding module (CBM) that can bind to the bacterial host in a way similar to that of the RBP (19, 20). In fact, it has been suggested that these evoDit proteins assist the RBP by helping to position the virion correctly and to ensure efficient receptor recognition and subsequent genome injection (20).

While the adsorption processes of model phages infecting B. subtilis (i.e., the siphovirus SPP1 and the podovirus Φ29) have been fairly well characterized, little information is available on the adsorption of tailed phages targeting Bacillus cereus (11, 12, 21). Indeed, for tailed phages infecting members of the B. cereus group (i.e., commonly accepted members are B. cereus sensu stricto, Bacillus thuringiensis, Bacillus anthracis, Bacillus weihenstephanensis, Bacillus cytotoxicus, Bacillus mycoides, and Bacillus pseudomycoides), the receptor has only been identified for the siphophage gamma, which is specific to B. anthracis and adsorbs to a surface protein called GamR. Its RBP remains so far unknown (22).

In this work, we first present a detailed analysis of the tail proteins (TPs) encoded by siphoviruses infecting members of the B. cereus group (also known as B. cereus sensu lato). This analysis revealed several distinct tail organizations and the presence of CBMs in various TP types (i.e., Dit protein, RBPs, and Tal), suggesting their importance for phage adsorption. The specific and differential implications of both candidate proteins RBP and evoDit in the adsorption of phage Deep-Purple (23) were then experimentally investigated. We show that the sole adsorption of evoDit is not sufficient for a successful infection but significantly contributes to the phage’s adsorption to its carbohydrate receptor(s).

## RESULTS

### Siphoviruses infecting members of B. cereus sensu lato display five distinct tail organizations.

**FIG 1:**
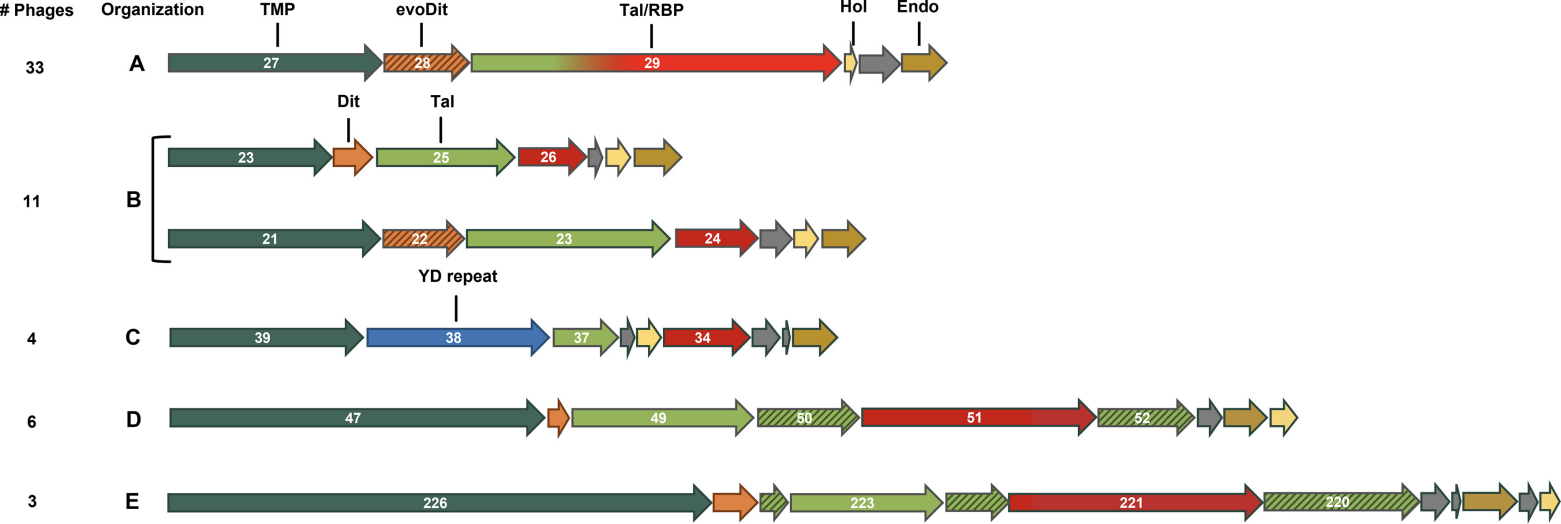
Genetic organization of putative tail genes in *Siphoviridae* infecting members of the B. cereus group. Five different gene syntenies are encountered (A to E), and the numbers of phages displaying such organizations are indicated on the left. The tail modules were retrieved from phages Deep-Purple (A), vB_BtS_BMBtp13 (top), and vB_BceS-IEBH (bottom) (B), Carmen17 (C), Basilisk (D), and vB_BanS-Tsamsa (E). TMP (dark green), tape measure protein; Dit (orange), classical distal tail protein; evoDit (striped orange), evolved Dit protein; RBP (red), receptor binding protein; YD repeat (blue), tyrosine-aspartate dipeptide repetitive unit protein; Hol (yellow), holin; Endo (brown), endolysin; Tal (green), tail lysin. Other tail genes are indicated in striped green, and hypothetical genes are in gray. Numbers inside arrows refer to the gene numbers used for each phage.

The genetic organization of regions encoding tail proteins (TPs) of 57 siphoviruses infecting members of the B. cereus group was analyzed (Fig. 1; see Table S1 in the supplemental material). As for most *Siphoviridae* phages, TPs are encoded between the tape measure protein (TMP) gene and the lysis cassette (i.e., holin and endolysin), with the major tail protein (MTP) upstream gene *tmp* usually separated by several chaperone genes (1). However, a detailed comparison revealed the existence of five distinct genetic organizations of the tail morphogenesis module (Fig. 1A to E).

**FIG 2:**
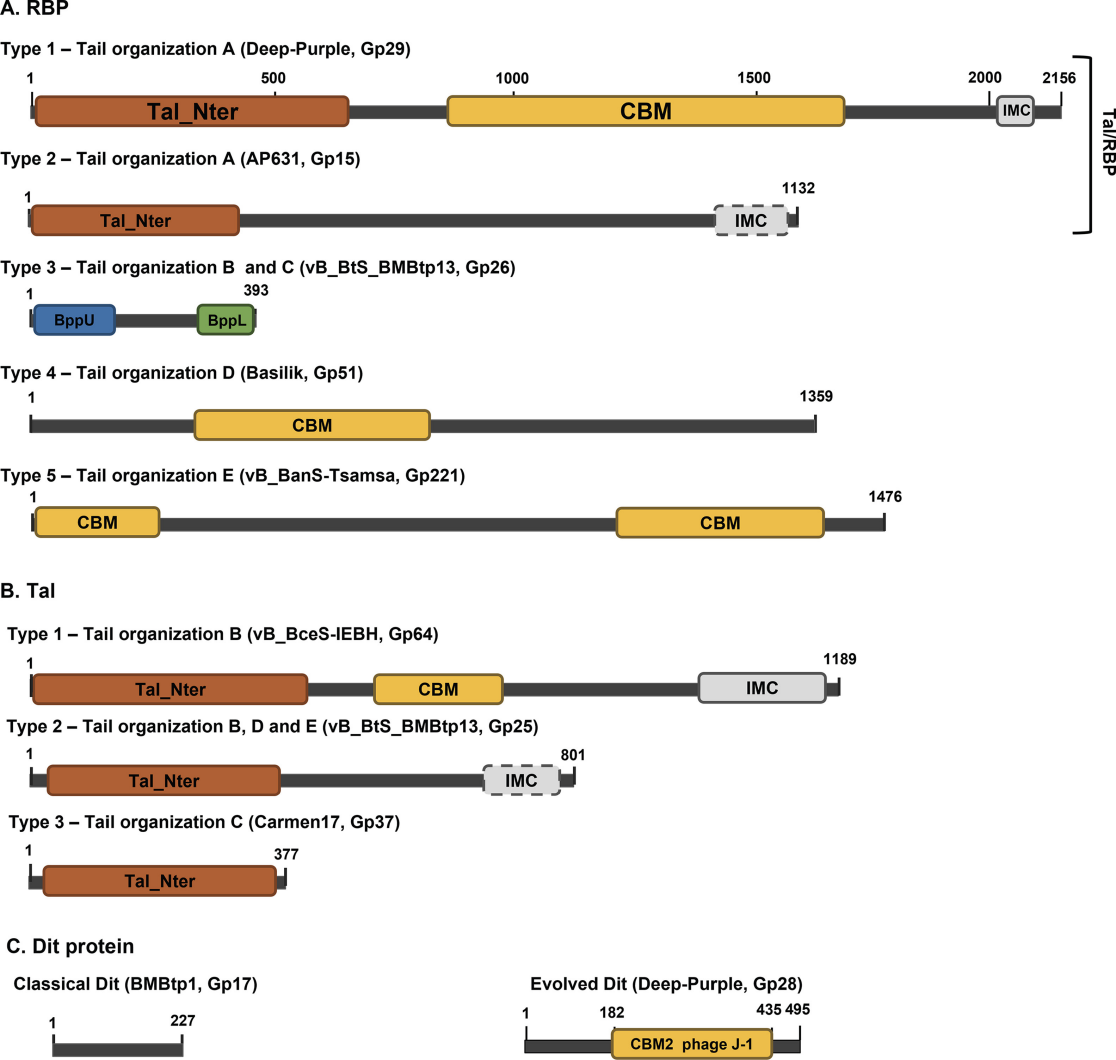
Domain organization of RBP, Tal, and Dit proteins found in siphoviruses infecting members of the B. cereus group based on HHpred analysis. Five types of RBP (A), three types of Tal protein (B), and two types of distal tail (Dit) protein (C) have been identified in the different tail loci (see Fig. 1 for details). An example is depicted for each protein type, with the corresponding phage and gene product (Gp) indicated between brackets. A dashed line indicates that the domain is not present in all phages displaying the tail organization. Tal_Nter, N-terminal domain of Tal; CBM, carbohydrate binding module; IMC, intramolecular chaperone; BppU, upper baseplate protein domain; BppL, lower baseplate protein domain.

**FIG 3:**
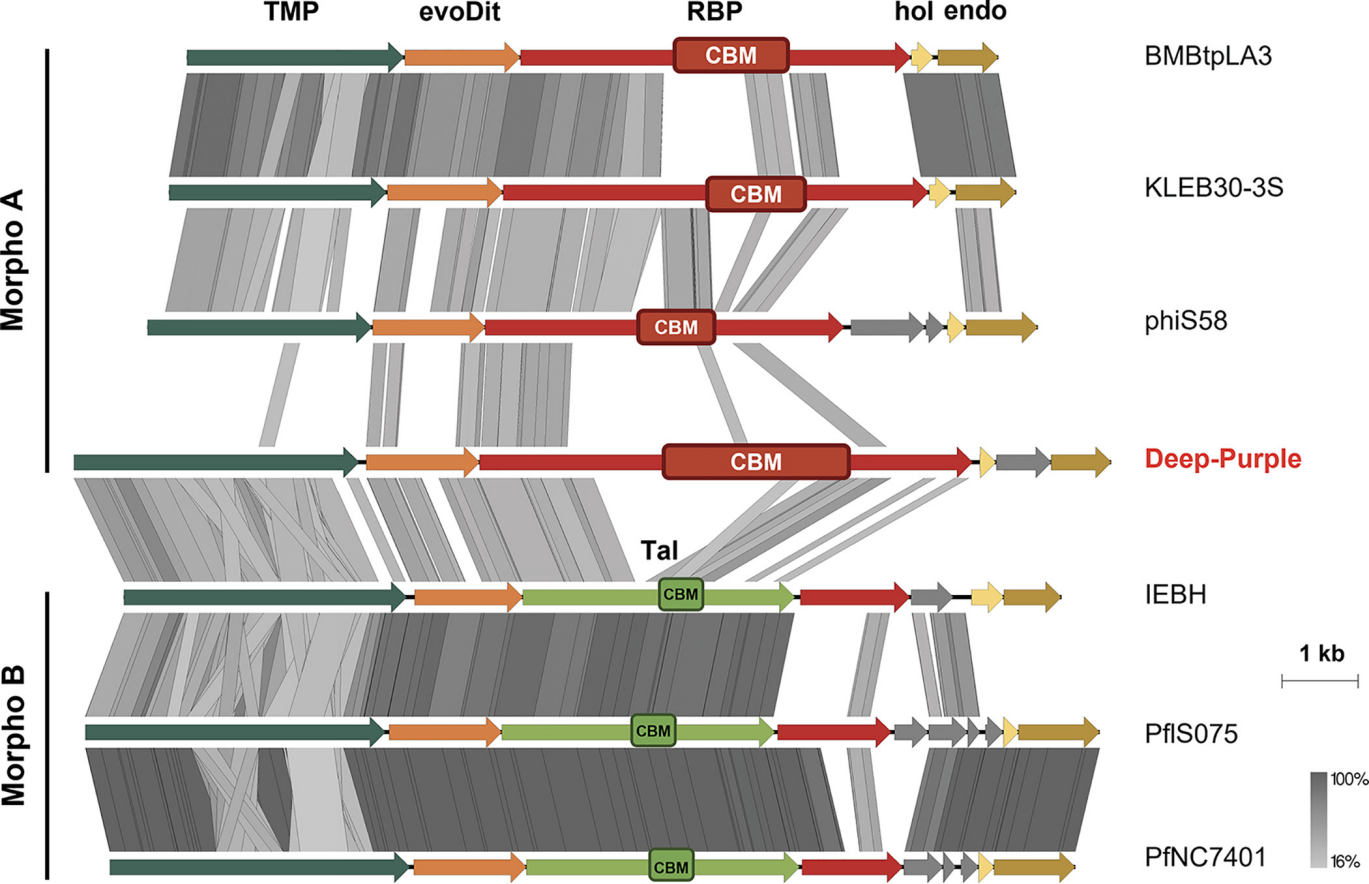
Comparison of the tail morphogenesis module of Deep-Purple with those of phages displaying organization A or B (Fig. 1). CBMs are indicated for RBP and Tal but were omitted for evoDit proteins for the sake of clarity. Deep-Purple is highlighted in red. The different comparisons were generated with Easyfig using tBLASTx. The scales of the genetic loci (in kb) are located on the right, together with the gradient (gray) scale of protein identity. Color codes and acronyms are as in Fig. 1 and 2.

Genetic organization A is the most prevalent among siphophages investigated in this study (i.e., 33/57), including phage Deep-Purple (see below). Similar to the tail morphogenesis module of B. subtilis phage SPP1, it consists of the *tmp* gene, followed by the *dit* gene and a large *tal* gene that presumably encodes the RBP (11). The large sizes of Tal/RBP (1,098 to 2,156 aa), combined with the presence of coiled-coil segments, suggest that they are putative tail fibers or spikes (24). Similar to phage SPP1, no PG-degrading domain was identified in Tal/RBP. Their N-terminal regions display structural homologies with the Tal protein of S. aureus phage 80α (Protein Data Bank [PDB] ID 6V8I_AE) and Gp18, a TP of L. monocytogenes prophage EGD-e (PDB ID 3GS9_A) (see Table S2 in the supplemental material) (25). Besides, for most Tal/RBP proteins, an intramolecular chaperone (IMC) domain (InterPro ID IPR030392) was detected at their C-terminal extremity (Table S1). IMCs are commonly found in fibers and spikes, in which they assist their trimerization before releasing themselves upon the correct folding (26–28). More importantly, the central part of the RBP sequences can be of two types (Fig. 2A and Fig. 3A). On the one hand, the presence of a CBM corresponding mainly to the xylan-binding CBM22-1–CBM22-2 tandem domain (PDB ID 4XUP_D) was detected in 14 phages, suggesting they may depend on polysaccharides for adsorption (Fig. 2A, type 1). On the other hand, no CBM was detected in the other 19 phages (Fig. 2A, type 2), including the gamma phage infecting B. anthracis. It is noteworthy that the receptor recognized by this phage is GamR, a surface protein (22).

Genetic organization B (11/57 phages) displays a similar synteny to that of many L. lactis phages (e.g., p2), with three TPs encoded downstream of the *tmp* gene in the following order: Dit protein (classical or evolved), Tal, and RBP (18, 29) (Fig. 1B). Their Tal N-terminal parts exhibit structural similarities to those of S. aureus 80α, while their C-terminal parts usually contain an IMC (Fig. 2B). Similar to phage p2, which cannot infect L. lactis when the PG is highly cross-linked (i.e., during the stationary phase), no PG degradation domain was identified in the Tal sequences (29). Interestingly, some Tal proteins (*n *= 4) present a CBM in the central part of the protein sequence, which was also observed in Streptococcus thermophilus siphoviruses (Fig. 2B, type 1) (30). The last TP is the putative RBP that displays an organization similar to that of L. monocytogenes phage PSA (Fig. 2A, type 3) (31). The N-terminal end is similar to the upper baseplate protein of phage TP901-1 (BppU; PDB ID 4V96_AA) involved in the RBP attachment to the Dit hexamer, whereas the C-terminal part matches phage TP901-1 lower baseplate protein (BppL; PDB ID 4IOS_A), which is responsible for host recognition (32).

Genetic organization C (4/57 phages) is quite atypical, as no Dit-like protein could be identified, although this protein is usually well conserved among *Siphoviridae* infecting both Gram-positive and Gram-negative bacteria (33) (Fig. 1C). Instead, the *tmp* gene is directly followed by a gene encoding a putative YD repeat protein (referring to a tyrosine-aspartate dipeptide repetitive unit) known to be involved in carbohydrate binding, suggesting that this protein may play a role in adsorption (34). The second TP is a putative type 3 Tal (Fig. 2B; Table S2) (25). Interestingly, the *rbp* gene, which is similar to that found in organization B, is not located in the tail structural module but is either upstream of the *mtp* gene (e.g., phage Anath) or directly after the holin gene (e.g., phage PBC1) (Fig. 1C).

Organizations D and E are found in *Siphoviridae*, with large genomes of *ca*. 80 kb (6/57 phages) and 160 kb (3/57 phages), respectively (Fig. 1D and E). These phages have complex tail modules with more TPs than in other siphoviruses analyzed here (35, 36). A classical Dit protein is encoded directly downstream of the *tmp* gene, followed by four to five TP genes. Among these TP genes, two tail fibers are encoded: a type 2 Tal and the putative RBP. The latter harbors either a central CBM (Fig. 2A, type 4) or two CBM regions located at the N- and C-terminal ends (Fig. 2A, type 5). The function of the other TPs is unknown, but some possess Ig-like folds which have been previously suggested to be involved in interactions with carbohydrates to improve phage adsorption (Table S2) (37, 38).

### evoDit proteins are widespread among siphoviruses targeting the B. cereus group.

Further analysis of the Dit protein structural organization revealed two distinct types among the siphoviruses infecting the B. cereus group: “classical” and “evolved” (also known as evoDit) (Fig. 2C). The classical Dit proteins were identified in 15 phages with tail organizations B, D, and E (Fig. 1). Those encoded by phages with tail organizations B and E are homologous and have similar sizes (i.e., ~250 to 300 aa) to the classical Dit proteins observed in phages infecting L. lactis (Table S2) (20), while those with tail organization D are smaller (121 aa) and have structural homologies with that of Escherichia coli phage T5 (PDB ID 6F2M_C).

evoDit proteins (383 to 504 aa in length) are more prevalent (*n *= 38) and were found in all phages with tail organization A and a few with organization B (Table S1). The evoDit proteins are similar to classical Dit proteins in their N and C extremities but bear a central domain homologous to CBM2 of *Lactobacillus* phage J-1 evoDit protein (PDB ID 5LY8_A) (Fig. 2C). The structure of this CBM has recently been resolved, and it was shown to bind to Lactobacillus casei cell wall polysaccharides (19). Interestingly, though, some evoDit proteins are atypical. For instance, in phages phBC6A51 and vB_BtS_BMBtp2, the CBM is located at the N-terminal part, and no CBM could be detected in the large (643-aa) Dit protein of phage vB_BthS_BMBphi (organization B).

### The Deep-Purple adsorption locus displays the typical arrangement of tail organization A.

Deep-Purple is a 36-kb siphovirus that shares almost no DNA sequence identity with other phages (23). The genetic organization of its TP belongs to tail morphogenesis A with a putative Dit protein (Gp28) and RBP (Gp29) harboring central CBM regions (Fig. 1A and Table 1). Gp28, the potential evoDit protein (495 aa), possesses a 254-aa CBM (coordinates 182 to 435) corresponding to the CBM2 domain of *L. casei* phage J-1 evoDit (Fig. 2 and Table 1). The Deep-Purple putative RBP, Gp29, has a size of 2,156 aa, which is larger than what is usually observed in other RBPs of tail morphogenesis A (i.e., 1,100 to 1,800 aa). The protein organization is typical of the three-domain arrangement found in type 1 RBP (Fig. 2A and Table 1). The central part of Gp29 is occupied by a ca. 800-aa region exhibiting structural similarities to several CBMs, including that of a xylanase (Xyn10C) encoded by Paenibacillus barcinonensis (4XUP_D), which is in fact constituted of three individual CBMs found in tandem repeats (Table 1; see Fig. S1 in the supplemental material). Comparison of Deep-Purple TP with those of phages from organizations A and B shows that the N-terminal part of Gp29 is conserved, whereas the central CBM region and, to a lesser extent, the C-terminal part of the protein are more variable (Fig. 3). Similarly, the central part of the evoDit-Gp28, corresponding to the CBM region, appears to be less conserved.

**TABLE 1:** Relevant hits corresponding to the HHpred analysis of Deep-Purple evoDit protein (Gp28) and RBP (Gp29)^*a*^

Protein (size in aa)	Residues (aa)	Match	PDB ID	Prob (%)	%Id
evoDit-Gp28 (495)	182–435	Lactobacillus casei phage J-1 evolved Dit CBM2, Gp16	5LY8_A	100	18

	1–192	B. subtilis SPP1 Dit protein, Gp19.1	2X8K_A	99.8	19
	434–495			98	24

	1–199	Lactococcus lactis phage TP901-1 Dit protein, orf46	4V96_AV	99.6	14
	434–495			98	15

	1–201	S. aureus phage 80α Dit protein, Gp58	6V8I_CC	99.6	15
	416–495			97.3	21

	38–192	L. lactis phage p2 Dit protein, orf15	2WZP_Q	97.7	11
	434–494			92.4	14


RBP-Gp29 (2,156)	7–457	S. aureus phage 80α Tal, Gp59	6V8I_AE	100	13

	4–383	Listeria monocytogenes EGD-e prophage TP, Gp18	3GS9_A	99.9	12

	17–357	Shewanella oneidensis prophage MuSo2 tail protein	3CDD_F	98.3	11

	868–1165	Paenibacillus barcinonensis Xyn10C CBM22-1–CBM22-2 tandem domain	4XUP_D	96.5	11
	1013–1374			97.3	12
	1206–1639			97.5	11

	809–1165	CBM of endo-α-*N*-acetylgalactosaminidase from S. pneumoniae	3ECQ_B	95.3	8
	1075–1374			94.5	9
	1211–1643			95.7	7

	811–1164	CBM of insecticidal protein Vip3Aa from B. thuringiensis	6TFJ_B	93.4	9
	1075–1373			92.2	9
	1211–1638			93.6	9

	2016–2137	E. coli phage K1F endo-*N*-acetylneuraminidase IMC	3GW6_A	97.1	17

	2016–2133	E. coli phage T5 L-shaped tail fiber with its IMC domain	4UW8_A	96.8	12

a The location of the HHpred hit in the protein is indicated in the “Residues” column. PDB ID, Protein Data Bank number; Prob, probability of the HHpred hit; %Id, percentage of amino acid identity.

### Deep-Purple evoDit-Gp28 and RBP-Gp29_CBM differentially bind to strains of the B. cereus group.

To investigate the possible involvement of the putative evoDit-Gp28 and RBP-Gp29 in Deep-Purple adsorption, both proteins were fused to a green fluorescent protein (GFP) tag in order to perform cell wall decoration assays (see Fig. S2 in the supplemental material). Since purification of the GFP fusion to the entire RBP-Gp29 protein could not be achieved, only a fusion of its CBM to GFP (RBP-Gp29_CBM-GFP) was further used. Regarding the tested bacteria, the B. cereus group strains were selected based on their differential sensitivity to Deep-Purple. Besides the usual sensitive or insensitive strains, some display an intermediate sensitivity phenotype characterized by lysis only at high phage concentrations. This bactericidal activity does not lead to the production of new virions (characterized by the observation of lysis plaque) and can be explained by different phenomena, including lysis from without (i.e., premature lysis of bacteria mediated by tail lysins without phage multiplication) (39) or abortive infection resistance mechanisms (i.e., bacterial cell “suicide” to prevent phage multiplication and infection of neighboring bacteria) (40).

**FIG 4:**
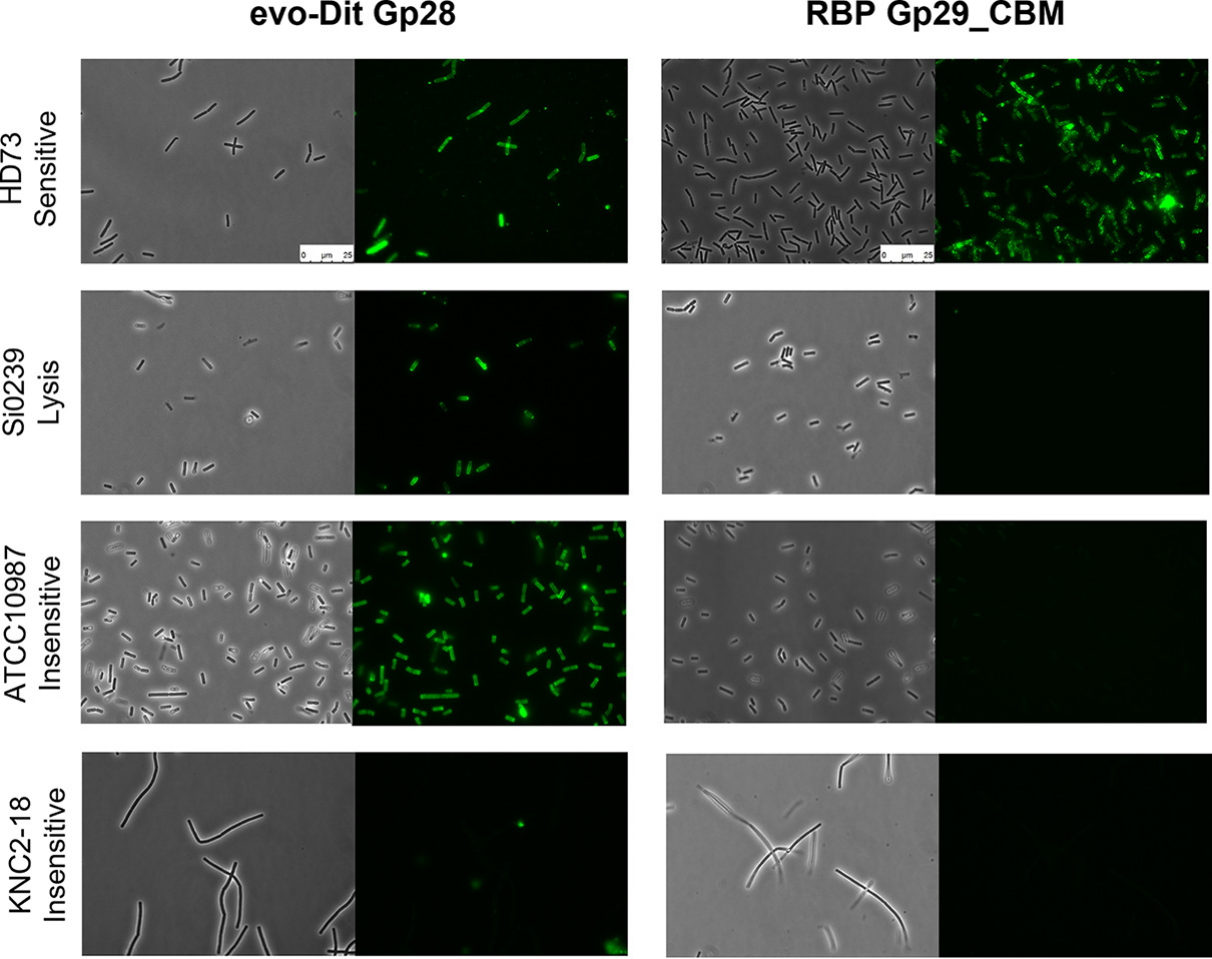
Cell wall binding assay of evoDit-Gp28 and RBP-Gp29_CBM to bacteria of the B. cereus group. evoDit-Gp28 and RBP-Gp29_CBM were fused to a GFP tag (Fig. S2) to assess their binding abilities. Exponentially growing *Bacillus* cells of the four strains, either sensitive (HD73), insensitive (KNC2-18 and ATCC 10987) or affected by lysis (Si0239), were incubated with ca. 10 to 20 μg of evoDit-Gp28::GFP (left panel) or RBP-Gp29_CBM::GFP (right panel) and observed using an epifluorescence microscope. The first and third columns show phase-contrast microscopy images, whereas the second and fourth columns refer to the corresponding fluorescent images. The scale is identical for all images.

Results obtained for the cell wall decoration assay are summarized in Table 2 and illustrated in Fig. 4 (left panels). evoDit-Gp28 was able to recognize all sensitive strains (*n *= 4) as well as those affected by lysis (*n *= 11). Regarding the strains insensitive to Deep-Purple infection (*n *= 7), only two were recognized by Gp28. The CBM of Gp28 was also expressed in fusion with GFP and gave the same results as those obtained with the whole protein, which confirms the implication of CBM in the binding of the fusion protein to the bacteria (Table 2; see Fig. S3 in the supplemental material). Concerning, Gp29_CBM, it only recognized the sensitive strains, and none of the insensitive strains or those affected by lysis could be decorated by the GFP tagged protein (Fig. 4, right panels).

**TABLE 2:** Binding spectra of evoDit-Gp28, evoDit-Gp28_CBM, and RBP-Gp29_CBM^*a*^

Strain	Reference or source^*b*^	Deep-Purple sensitivity	Binding spectrum of:		
			evoDit-Gp28	Gp28_CBM	RBP-Gp29_CBM
B. cereus					
ATCC 10987	65	I	+	+	−
H3081.97	66	L	+	+	−
TIAC139	Sciensano	L	+	+	−
VD021	67	S	+	+	+

*B. cytotoxicus*					
E17.4	68	L	+	+	−
NVH 391-98	INRAE	L	+	+	−
PDT2.12	68	L	+	+	−
SM1.1	68	L	+	+	−
SM2.8	68	L	+	+	−

B. mycoides					
KNC2-18	MIAE	I	−	−	−

B. thuringiensis					
AW43	69	S	+	+	+
DBT242	MIAE	I	+	+	−
HD73	BGSC	S	+	+	+

B. weihenstephanensis					
BtB2-4	70	L	+	+	−
KBAB4	71	I	−	−	−
LH002	23	S	+	+	+
MC67-2	72	I	−	−	−
MC118-4	72	I	−	−	−
SI0170	MIAE	I	−	−	−
SI0239	73	L	+	+	−
WSBC10202	74	L	+	+	−
WSBC10204	74	L	+	+	−

a The GFP-fused proteins were tested on various strains of the B. cereus group in a cell wall decoration assay. The “Deep-Purple sensitivity” column indicates the bacterial sensitivity to the phage: I, insensitive; S, sensitive; and L, lysis. Binding and nonbinding of the corresponding proteins to the different strains is indicated by “+” and “−” respectively.

b The following sources are shown: Sciensano, Belgian Institute for Health, Brussels, Belgium; INRAE, Institut National de Recherche pour l’Agriculture, l’Alimentation et l’Environnement, Jouy-en-Josas, France; MIAE, Food and Environmental Lab, Université catholique de Louvain, Louvain-la-Neuve, Belgium; and BGSC, *Bacillus* Genetic Stock Center, Ohio State University, Columbus, OH, USA.

**FIG 5:**
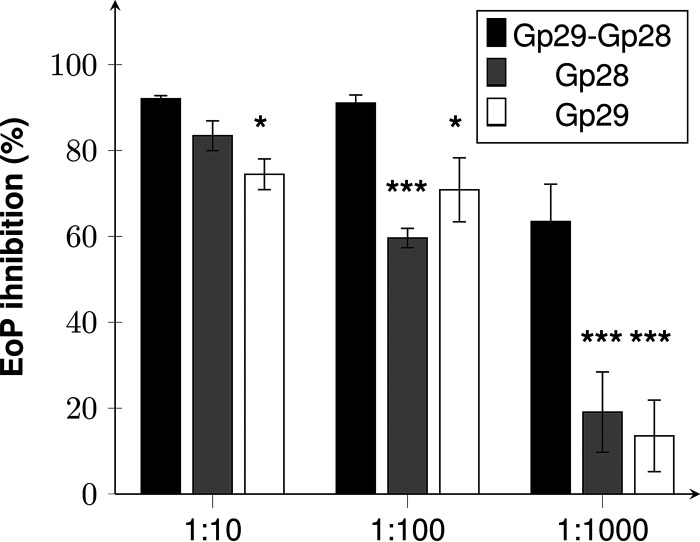
Competition assay using anti-Gp28 and anti-Gp29 sera. Deep-Purple phages were challenged with decreasing concentrations of antibodies raised against evoDit-Gp28 (gray), RBP-Gp29_CBM (white), or both types of antibodies (black). The efficiency of plating (EoP) of the phage was then assessed, and the results are expressed as inhibition percentage of the EoP compared to the EoP of untreated phages. Standard deviations were obtained from three independent replicates, and statistical differences from the condition where both antibodies were used are indicated with asterisks: *, *P* < 0.05; ***, *P* < 0.001.

In order to further validate these observations, antibodies were raised against evoDit-Gp28 and RBP-Gp29_CBM, and their effect on Deep-Purple efficiency of plating (EOP) was evaluated. As shown in Fig. 5, both anti-Gp28 and anti-Gp29_CBM sera reduced the phage EOP in a dose-dependent manner. Interestingly, Deep-Purple EOP was far more impacted when both antibodies were used simultaneously (in particular at a dilution of 1:1,000). These observations could be an indication of a complementary role of both proteins in Deep-Purple adsorption.

### Differences in Deep-Purple adsorption abilities to B. cereus sensu lato strains are observed.

**FIG 6:**
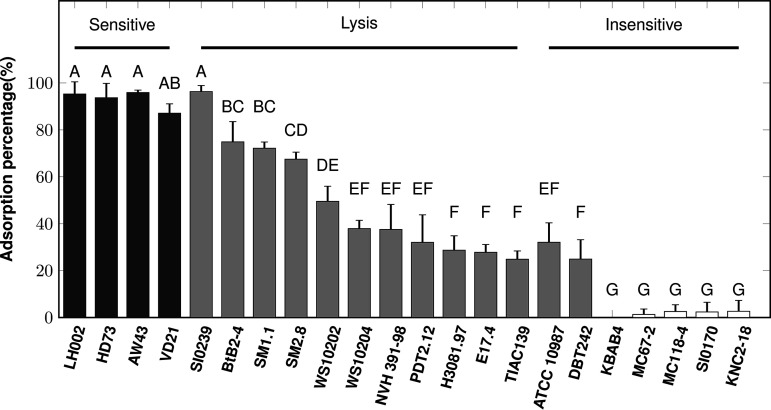
Deep-Purple adsorption to strains of the B. cereus group. An adsorption assay was performed to assess differences in the adsorption ability of Deep-Purple on different strains of the B. cereus group (see Table 2 for strain details). Black bars, strains recognized by both the RBP-Gp29 and the evoDit-Gp28; gray bars, strains recognized only by evoDit-Gp28; white bars, strains recognized by none of the proteins. Standard deviations were obtained from three independent replicates. The brackets indicate the strain sensitivity to Deep-Purple phage. Bars sharing the same letter are not statistically different from each other based on Tukey’s law (α = 0.05).

To better assess the relationship between Deep-Purple host spectrum and bacterial recognition, its adsorption capacity was tested on a range of B. cereus sensu lato strains displaying different sensitivities to the phage. As shown in Fig. 6, three situations were observed. A first cluster gathers strains to which Deep-Purple can adsorb with a high efficiency (i.e., more than 85%) and is represented by all of the sensitive strains. These strains were shown to be recognized by both evoDit-Gp28 and RBP-Gp29_CBM in the cell wall decoration assay (Table 2). Interestingly, Deep-Purple displayed a high adsorption ability for strain Si0239 (B. weihenstephanensis), which was affected by lysis and was only recognized by evoDit-Gp28. In the second group, Deep-Purple particles adsorbed with a moderate efficiency (i.e., 20 to 80%). These strains include those that are decorated by only evoDit-Gp28 in the cell wall decoration assay, namely, those affected by lysis and two insensitive strains (i.e., ATCC 10897 and DBT242). This observation supports that the evoDit-Gp28 should be implicated in phage adsorption. Finally, Deep-Purple particles did not adsorb to the insensitive strains that are recognized by neither the RBP nor evoDit. Overall, both RBP-Gp29 and evoDit-Gp28 should be involved in adsorption, although to different extents. The results are compatible with RBP-Gp29 being mandatory for infection, while the binding of evoDit-Gp28 alone does not seem sufficient to support successful infection.

### The Deep-Purple receptor is likely to be of a carbohydrate nature.

**FIG 7:**
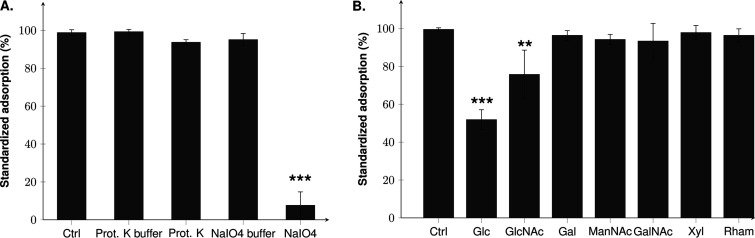
Assessment of the Deep-Purple receptor nature. (A) B. thuringiensis AW43 cells were treated with either proteinase K, to alter proteins, or sodium periodate, to alter carbohydrates. (B) Competition assays were performed by incubating Deep-Purple phages with different sugars followed by an adsorption assay with the sensitive strain (B. thuringiensis AW43). Results were derived from three independent experiments and standardized compared to the highest adsorption rate, which was set to 100% for each experiment. Statistical analyses are based on Tukey’s law, and asterisks indicate statistical differences: **, *P* < 0.01, ***, *P* < 0.001. Ctrl, control (no treatment or no sugar added); Glc, glucose; GlcNAc, *N-*acetylglucosamine; Gal, galactose; ManNAc, *N*-acetylmannosamine; GalNac, *N*-acetylgalactosamine; Xyl, xylose; Rham, rhamnose.

*In silico* analysis of evoDit-Gp28 and RBP-Gp29 revealed that they both have CBM, suggesting that Deep-Purple depends on carbohydrate structures for adsorption. In order to assess the involvement of proteins and/or carbohydrates in Deep-Purple adsorption, the host strain B. thuringiensis AW43 was treated with either proteinase K or sodium periodate. As illustrated in Fig. 7A, alteration of the host carbohydrates decreased Deep-Purple adsorption by ca. 90% compared to the control treatment, whereas the proteinase K treatment had no impact, suggesting that the phage exclusively relies on carbohydrate structures for its adsorption.

To further document the sugars that could be involved in the recognition/adsorption process, a competition assay was performed using various sugars found in secondary cell wall polysaccharides of B. cereus (glucose, *N*-acetylglucosamine, galactose, *N*-acetylgalactosamine and *N*-acetylmannosamine) (41). Sugars identified as binding ligands of the structural homologues of evoDit-Gp28 (rhamnose) and RBP-Gp29 (xylose) were also tested (19, 42). The use of glucose and *N*-acetylglucosamine induced strong interference with the adsorption of Deep-Purple to strain AW43, which was reduced by, respectively, ca. 48 and 23% compared to its normal adsorption rate (Fig. 7B). The other tested sugars did not significantly affect Deep-Purple adsorption abilities. It is noteworthy that glucose and *N-*acetylglucosamine substitution on wall teichoic acids are known to be implicated in the adsorption of several phages infecting Gram-positive hosts, notably B. subtilis, S. aureus, and L. monocytogenes (43–45).

### Both evoDit-Gp28 and RBP-Gp29 are located at the tip of the Deep-Purple baseplate.

**FIG 8:**
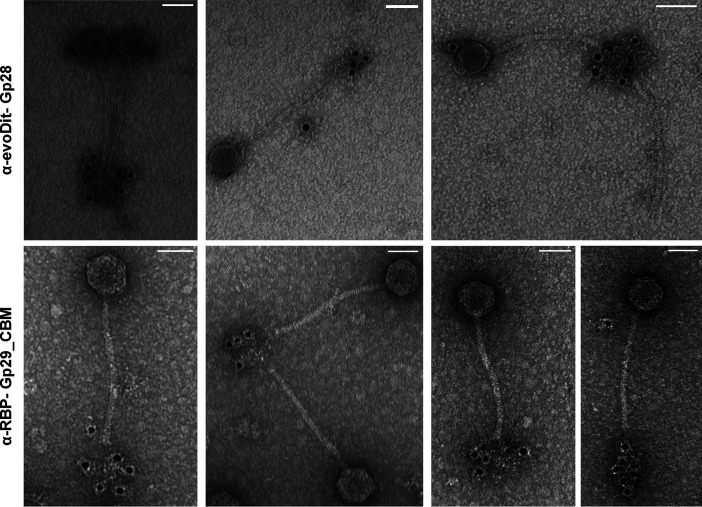
Localization of evoDit-Gp28 and RBP-Gp29_CBM proteins in Deep-Purple virions by immunogold electron microscopy. Antibodies were raised against evoDit-Gp28 (upper panels) and RBP-Gp29_CBM (lower panels) and used for localization of the related proteins using secondary antibodies coupled to gold nanoparticles. Scale bars correspond to 50 nm.

Transmission electron microscopy (TEM) combined with immunogold labeling was performed to locate the evoDit-Gp28 and RBP-Gp29_CBM in the Deep-Purple virion. Briefly, Deep-Purple was incubated with antisera containing antibodies raised against each protein, followed by localization using secondary gold-labeled antibodies. As predicted by the *in silico* analysis, evoDit-Gp28 and RBP-Gp29 are located at the baseplate (Fig. 8). As observed for phage J-1 infecting *L. casei*, evoDit-Gp28 is present in several copies per virion and is located at the tip of the baseplate. As for the CBM of RBP-Gp29, several copies could also be detected at the baseplate of Deep-Purple particles. However, compared to evoDit-Gp28, RBP-Gp29 is apparently located further away from the baseplate, possibly in a structure extending the phage tail (i.e., tail fiber or spike).

## DISCUSSION

The analysis of the tail-encoding regions of siphoviruses targeting the B. cereus group highlighted distinct genetic organizations. The majority of phages exhibit two main gene syntenies, which are similar to those of the well-known phages SPP1 (tail organization A) and p2 (tail organization B), infecting B. subtilis and L. lactis, respectively. However, other less typical tail arrangements are also observed. *In silico* analysis also demonstrated that CBMs are extensively found in Dit proteins and Tal. Such “evolved” proteins have been shown to be prevalent in phages targeting lactococci and streptococci (20, 30, 46).

In this work, the proteins potentially implicated in the adsorption of the phage Deep-Purple were experimentally investigated. Deep-Purple encodes two baseplate proteins: Gp28, an evoDit-like protein, and Gp29, a large Tal protein encoded by a gene supposedly encoding the RBP function and containing a central CBM region. The evoDit-Gp28 and the CBM of RBP-Gp29 were therefore explored as adsorption-related proteins. The GFP-fused RBP-Gp29_CBM showed the same selectivity as Deep-Purple and bound only sensitive strains. Conversely, evoDit-Gp28 was able to decorate lysis and insensitive strains, in addition to sensitive strains, suggesting that the adsorption of evoDit-Gp28 alone is not sufficient to trigger DNA injection. Instead, the adsorption of RBP-Gp29 to its cognate receptor may be the key interaction. It was previously suggested for phage J-1 infecting *L. casei* that the evoDit protein (Gp16) is in fact the *bona fide* RBP (19, 47). However, in this phage, the protein corresponding to the Tal/RBP of Deep-Purple could not be purified, and its binding abilities were thus not investigated (47). In fact, evoDit proteins are rather thought to be accessory proteins, assisting the RBP and involved in the initial reversible binding to bacteria, allowing the phage to be in close proximity to the bacterial surface to facilitate the search for the final receptor (48). In lactococcal phages, it was shown that the evoDit binding spectrum is identical to the phage host spectrum (20). The authors proposed that evoDit proteins bind to the same receptor as RBPs, but that CBMs, which are thought to be projected away from the baseplate, increase the possibilities of host attachment in any virion orientation. In the case of Deep-Purple, the wider binding spectrum of evoDit-Gp28 may suggest that these proteins are involved in the first reversible attachment, while the CBM present in RBP-Gp29, displaying an identical binding range to the phage itself, increases the chances to find and bind to the *bona fide* receptor.

In RBPs, the highly variable receptor binding domain is usually located at the C-terminal extremity of the protein, while the N-terminal part is more conserved and responsible for the protein anchorage in the baseplate (31, 49). In RBP-Gp29, the N-terminal part shows structural similarities to known Tal N-terminal parts, while the rest of the protein is far less conserved. In fact, two regions of low sequence identity with other homologous proteins were found: the central CBM region and the extreme C-terminal part (Fig. 3). Unfortunately, the C-terminal part of RBP-Gp29 could not be purified, and its binding abilities could not be assessed. However, due to the similarity to the RBP of B. subtilis phage SPP1 (i.e., Gp21), we hypothesize that the RBP-Gp29 C-terminal part should be involved in the event of receptor attachment leading to the triggering of genome injection and not the CBM region.

The adsorption of Deep-Purple was found optimal for strains recognized by both evoDit-Gp28 and RBP-Gp29_CBM. Nonetheless, the sole binding of evoDit-Gp28 correlates with a significant phage adsorption, strengthening the fact that this protein’s ability to recognize some structure at the bacterial surface improves adsorption. Interestingly, Deep-Purple was able to absorb with a high efficiency to B. weihenstephanensis Si0239, although this strain was only recognized by evoDit-Gp28. This might be explained by an unusual high density of evoDit-Gp28 ligand, compared to those of other *Bacillus* strains, thereby compensating for the lack of RBP-Gp29-recognized receptor.

Investigation of the receptor’s nature suggests that Deep-Purple relies on carbohydrates for its adsorption, which is consistent with the identification of several CBMs in its TP and its narrow host range (limited to a few strains of the B. cereus group). Indeed, phages displaying narrow host ranges often rely on polysaccharide receptors as they are highly variable and often strain specific (50). Given that evoDit-Gp28 and RBP-Gp29_CBM have different binding spectra, we hypothesize that they recognize different carbohydrate moieties, the one associated with evoDit-Gp28 presumably more prevalent among strains of the B. cereus group. Furthermore, the folds identified in both proteins were different as evoDit-Gp28 exhibits structural similarities to the CBM2 found in the evoDit of *L. casei* phage J-1, whereas various CBM folds carried, for instance, by bacterial xylanases or endo-α-*N*-acetylgalactosaminidases were detected in RBP-Gp29 (42, 51). The carbohydrate moieties present in the B. cereus group have not been extensively studied, but possible receptors could involve teichoic acid (i.e., cell wall teichoic acid and lipoteichoic acids) or nonclassical secondary cell wall polysaccharides (SCWPs) (52). Interestingly, the structure of these SCWPs was elucidated in two strains of B. cereus, and it was shown that they both possess a common backbone consisting of a trisaccharide repetitive unit, (→4)GlcNAc(β1–6)GalNAc(α1–4)ManNAc(β1→), but differ by their sugar substitutions (41, 53).

Deep-Purple has the same tail module genetic organization as that of phage SPP1, in which the large Tal/RBP harbors the receptor binding domain and forms the central spike involved in the recognition of the YueB protein (21, 54). Given that protein-protein interactions are strong, phages relying on the protein receptor possess a single copy of their trimeric RBP (55). In contrast, phages recognizing carbohydrate-based receptors have multiple RBP copies to compensate for the weak affinity between carbohydrates and proteins (13, 56). In Deep-Purple, the virion is expected to harbor a central tail spike formed by RBP-Gp29. Yet, our experiments suggest that Deep-Purple exclusively relies on carbohydrates for its adsorption and thus is expected to require multiple binding events to ensure a tight interaction with its host. Although the TEM experiments did not allow us to distinguish a central tail spike, the antibodies raised against RBP-Gp29_CBM bound the virions slightly away from its baseplate, suggesting the presence of such structure. Thus, it may be possible that Deep-Purple harbors a single central spike interacting with the carbohydrate receptor but that the presence of a CBM in both the Dit protein and the RBP allows it to increase the phage adsorption. Interestingly, in RBP-Gp29 the CBM region is in fact composed of three repeats of individual CBMs, and it is known that CBMs are often found in tandem repeats to increase their binding affinity (57).

In conclusion, our results showed that phage Deep-Purple depends on both an evoDit protein and an RBP for efficient binding to its B. cereus hosts, an adsorption that relies on carbohydrates. The *in silico* analysis of phages infecting the B. cereus group also indicates that their tail structures and the adsorption mechanisms are diverse, displaying both similarities to and differences from those currently described in phages infecting other Gram-positive bacteria.

## MATERIALS AND METHODS

### Bacterial strains, plasmids, and growth conditions.

The bacterial strains and plasmids used in this work can be found in Tables 2 and 3. Bacteria were grown in a lysogeny broth (LB) or LB agar plate at 30°C for B. cereus and 37°C for E. coli. When required, kanamycin (selection for pET30 plasmid) and/or chloramphenicol [Rosetta(DE3)/pLysS] (Sigma, Saint-Louis, MO, USA) was added to the plates or liquid cultures at a final concentration of 50 μg/mL.

**TABLE 3:** Strains and plasmids used in cloning experiments

Strain or plasmid	Species or purpose	Source^*a*^
Strains		
10-beta	E. coli	NEB
BL21(DE3)	E. coli	Merck
Rosetta(DE3)/pLysS	E. coli	Merck

Plasmids		
pET30a	Expression vector	NEB
pUC18::*gfp*	GFP amplification	Clontech/TaKaRa
pET30::*gp28*	Expression of Gp28	This work
pET30::*gp28*(linker)::gfp	Expression of GFP-fused Gp28	This work
pET30::*gp28_cbm*(linker)::*gfp*	Expression of GFP-fused Gp28_CBM	This work
pET30::*gp29_cbm*	Expression of Gp29_CBM	This work
pET30::*gfp*(linker)::*gp29_cbm*	Expression of GFP-fused Gp29_CBM	This work

a The sources include New England BioLabs (NEB), Ipswich, MA, USA, Merck, Darmstadt, Germany, and Clontech/TaKaRa, Saint-Germain-en-Laye, France.

### Bioinformatic tools.

Bioinformatic analyses of the phage tail proteins were done by genome comparison using Mauve (58) and EasyFig (59), and protein sequence analysis was done using HHpred (60), Interpro (61), and BLASTp (62). Phage genomes, as well as Dit protein and RBP sequences, were retrieved from NCBI. Their accession numbers can be found in Table S1 in the supplemental material.

### Molecular cloning.

PCR amplifications of target genes were done using *ad hoc* primers (Table 4) and Q5 polymerase (New England Biolabs [NEB], Ipswich, MA, USA). The resulting amplicons and the expression vector pET30a were cleaved using appropriate restriction enzymes (NEB) and ligated with the T4 DNA polymerase (Promega, Madison, WI, USA). The constructs were transformed into competent E. coli 10-beta cells, and the positive clones were verified by sequencing (Macrogen, Amsterdam-Zuidoost, The Netherlands) and subsequently transformed into the expression strain BL21(DE3) or Rosetta(DE3)/pLysS (Merck, Burlington, MA, USA).

**TABLE 4:** Primers used in this study

Target	Name	Sequence (5′→3′)
*gfp* amplification for N-terminal fusion	GFP_EcoRI_F	TTCCGAATTCAAAGGAGAAGAACTTTTCACTGGAG
	GFP_EagI_linker_R	TTCGGCCGTCCACTACCTGATCCACTACCTTTGTAGAGCTCATCCATGCC

*gfp* amplification for C-terminal fusion	GFP_KpnI_linker_F	AAGGTACCGGTAGTGGATCAGGTAGTGGAAAAGGAGAAGAACTTTTCACTGGAG
	GFP_EagI_Stop_R	TACGGCCGTTATTTGTAGAGCTCATCCATGCC

*gp29_cbm* for N-terminal *gfp* fusion	gp29_CBM_EagI_F	TTCGGCCGGAGGTTTCGCAGTCTACTGTTA
	gp29_CBM_XhoI_R	TATTCTCGAGTTATGTATTCTCCATAAAAGCAGAAGGC

*gp29_cbm*	gp29_CBM_NcoI_F	TATCCATGGTTGAGGTTTCGCAGTCTACTGTTA
	gp29_CBM_XhoI_R	TATTCTCGAGTTATGTATTCTCCATAAAAGCAGAAGGC

*gp28* for C-terminal *gfp* fusion	gp28_BstBI_F	GGTTTTTCGAATCAACATTCAAATTCAACG
	gp28_KpnI_R	AAGGTACCCTTATAACGCTCCTTGTAAG

*gp28*	gp28_EagI_F′	TTCGGCCGATCAACATTCAAATTCAACG
	gp28_XhoI_R	TATACTCGAGTTACTTATAACGCTCCTTGT

*gp28_cbm* for C-terminal *gfp* fusion	gp28_CBM_BglII_F	TTAGATCTGTATTTTCGTTTAGGATATCCG
	gp28_CBM_KpnI_R′	TTGGTACCTTCTGTATTGATTCTTATTTTGTCCC

The whole Dit gene of phage Deep-Purple, *gp28*, was either cloned alone in pET30a or fused to a C-terminal *gfp* to yield pET30::*gp28* and pET30::*gp28*(linker)::*gfp*, respectively. Its putative carbohydrate binding module (CBM) region (aa 178 to 445) was cloned with a C-terminal *gfp* tag. The region of the RBP gene *gp29*, encoding the putative CBM (aa 800 to 1650), was cloned alone or with an N-terminal *gfp* gene, yielding pET30::*gp29_cbm* and pET30::*gfp*(linker)::*gp29_cbm*, respectively. All the GFP fusions (Fig. S2) comprise a short linker sequence (with seven alternating glycine and serine residues) between the GFP and the target proteins.

### Protein expression and purification.

Overnight (O/N) cultures of the expression strain E. coli BL21(DE3) or Rosetta(DE3)/pLysS containing the constructs were subcultured in fresh LB (dilution 1:20) and subsequently incubated at 37°C at 180 rpm until the optical density at 600 nm (OD_600_) reached 0.5 to 0.8, at which point 0.5 or 1 mM isopropyl-β-d-thiogalactopyranoside (IPTG) (Sigma, Saint-Louis, MO, USA) was added. The cultures were then incubated at 28°C for 6 h or at 22°C O/N. The bacteria were then harvested by centrifugation (4,000 × *g*, 15 min, 4°C) and stored at −20°C. For purification, the bacterial pellet was resuspended in lysis buffer (50 mM NaH_2_PO_4_ · H_2_O, 300 mM NaCl, 10 mM imidazole, 1 mg/mL lysozyme [pH 8]) supplemented with a protease inhibitor cocktail (1× Sigmafast inhibitor cocktail tablet, EDTA free; Sigma) and incubated at 10°C for 45 min. Three units per milliliter of Benzonase nuclease (Sigma) was added to reduce viscosity, and the soluble proteins were recovered by centrifugation at 10,000 × *g* for 30 min at 4°C. The clear lysate was filtered on 0.45-μm-pore filter (VWR, Oud-Heverlee, Belgium), and the protein was purified using Ni-nitrilotriacetic acid (NTA) columns (Qiagen, Hilden, Germany) following the manufacturer’s recommendations. The purity was assessed by SDS-PAGE (Bio-Rad, Hercules, CA, USA), and the protein concentration was established by a Bradford assay (63).

### Cell wall decoration assay.

A cell wall decoration assay was used to assess the binding of the GFP-fused proteins (8). In brief, 300 μL of exponentially growing bacteria (3 to 4 h) was recovered by centrifugation (10,000 × *g*, 5 min), washed twice with SM buffer (50 mM Tris-HCl, 100 mM NaCl, 8 mM MgSO_4_ [pH 7.5]), and resuspended in 100 μL of SM buffer. Ten to 20 μg of protein was added (the protein buffer and purified GFP were used as controls), and the suspension was incubated for 20 min at room temperature (RT). The suspension was then centrifuged (10,000 × *g*, 5 min at 4°C) and washed twice with cold SM buffer. Finally, bacteria were observed under an epifluorescence microscope (Leica AF6000) using a filter with an excitation wavelength ranging from 460 to 500 nm and an exposure time of 300 ms. Images were obtained using the Leica LAS AF software.

### Phage host spectrum and adsorption assay.

The phage host spectrum was established using the spot-on-plate method. In brief, 5 mL of soft agar (0.5% agar, 3 mM CaCl_2_, 3 mM MgSO_4_) was mixed with 100 μL of a 4-h culture of the given bacteria and poured onto an LB agar plate. After drying, 10-fold dilutions of Deep-Purple suspensions were spotted, and the plate was subsequently incubated at 30°C O/N. Three types of phage sensitivities were distinguished: strains that are sensitive (presence of individual lysis plaques), insensitive (no individual plaque or lysis area), or affected by lysis (presence of lysis area at high concentrations but not individual lysis plaques).

To assess the adsorption ability of Deep-Purple, O/N bacterial cultures were diluted 1:100 in fresh LB supplemented with 3 mM CaCl_2_ and 3 mM MgSO_4_ and incubated for 3 to 4 h. The culture OD_600_ was adjusted to 0.6 (ca. 10^6^ CFU/mL). Then, 950 μL of the bacterial culture was mixed with 50 μL of 10^6^ PFU/mL of Deep-Purple. Following incubation for 20 min at 30°C, the bacterial suspension was centrifuged at 10,000 × *g* for 10 min at 4°C to remove the bacteria and adsorbed phages. The supernatant was filtered on a 0.45-μm-pore filter, and the concentration of the nonadsorbed phages (PFU/mL) present in the supernatant was estimated by the double-layer agar assay, in which serial dilutions of the phage suspension were mixed with a sensitive strain and poured as a top layer on agar plates (64).

### Cell wall treatment and influence of various sugars on adsorption.

Five hundred microliters of an O/N culture of B. thuringiensis AW43 was collected by centrifugation (10,000 × *g* for 10 min at RT), washed with phosphate-buffered saline (PBS) (Sigma), and resuspended in 500 μL of either 100 mM NaIO_4_ (dilution buffer: 50 mM CH_3_COONa [pH 5.2]) or 0.2 mg/mL proteinase K (dilution buffer: 20 mM Tris-HCl, 100 mM NaCl [pH 7.5]). Controls were done using PBS and the respective dilution buffers. The NaIO_4_ treatments were incubated for 2 h at RT in the dark and the proteinase K treatments for 1 h at 45°C. The bacteria were then centrifuged (10,000 × *g* for 10 min at RT), and the pellet was washed twice with PBS and resuspended in 950 μL of LB (supplemented with 3 mM CaCl_2_ and 3 mM MgSO_4_) and 50 μL of phage suspension (ca. 10^6^ PFU/mL) to assess the efficiency of adsorption to the treated bacteria.

The impact of various sugars on Deep-Purple adsorption efficiency was tested by incubating 50 μL of phage suspension (ca. 10^6^ PFU/mL) with 50 μL of 500 mM sugar solution (glucose, *N*-acetylglucosamine, galactose, *N-*acetyl-d-mannosamine, *N-*acetyl-d-galactosamine, xylose, or rhamnose from Sigma) for 1 h at RT. Bacteria (900 μL of ca. 10^6^ CFU/mL) were then added to assess Deep-Purple adsorption as described above.

### Competition assay.

Polyclonal antibodies against Gp29_CBM and Gp28 were raised in mice by Eurogentec (Seraing, Belgium). To evaluate the effect of anti-Gp29_CBM and/or anti-Gp28 on efficiency of plating (EOP), 180 μL of phages (10^5^ PFU/mL) was mixed with dilutions of sera containing antibodies and incubated for 30 min at RT. The concentration of phages able to form plaques was then assessed by double-layer agar as previously explained.

### Immunogold electron microscopy.

Phages (ca. 10^10^ PFU/mL) were placed over a nickel grid for 20 s and incubated at RT for 1 h with primary antibodies (anti-Gp28 or anti-Gp29_CBM) diluted 1:100. The grid was washed in TGB buffer (20 mM Tris, 50 mM NaCl, 10 mM MgCl_2_) and incubated for 1 h with goat anti-mouse secondary antibodies conjugated with gold nanoparticles (10 nm) (Sigma) at a dilution of 1:50. Then the grid was washed with TGB buffer, and fixation was done with 0.25% (vol/vol) glutaraldehyde for 20 min at RT. Samples were stained with uranyl acetate for 30 s. Observations were made with a Tecnai 10 transmission electron microscope (Thermofisher, Merelbeke, Belgium), and images were captured with a Veleta charge-coupled device (CCD) camera.
