# Supplementary material for: Viral Proteins Involved in the Adsorption Process of Deep-Purple, a Siphovirus Infecting Members of the Bacillus cereus Group
Source: Appl Environ Microbiol. 2022 May 2;88(10):e02478-21. doi: 10.1128/aem.02478-21 (PMC9128512; doi:10.1128/aem.02478-21)
Supplement: SUPPLEMENTAL FILE 1 — Supplemental material. Download aem.02478-21-s0001.pdf, PDF file, 2.1 MB [file aem.02478-21-s0001.pdf]

Table S1: *B. cereus s.l.* siphophages classified based on the organization of their tail morphogenesis module. evoDit: Evolved Distal tail protein; CBM: Carbohydrate Binding Module; Tal: Tail lysin; RBP: Receptor Binding Protein; IMC: Intramolecular chaperone. "-" indicates that the protein is not present. The different genetic organizations are displayed in Fig. 1.

		Genome		Dit protein			Tal				RBP			
#	Phage	Accession #	Size (bp)	Accession #	Size (aa)	evo Dit	Accession #	Size (aa)	CBM	IMC	Accession #	Size (aa)	CBM	IMC
**Organization A**														
1	11143	GU233956.1	39077	ADA84945.1	491	Yes	-	-	-	-	ADA84942.1	1585	Yes	Yes
2	AP631	MK085976.1	39549	AZF88359.1	496	Yes	-	-	-	-	AZF88360.1	1332	No	Yes
3	BceA1	HE614282.1	42932	CCE73853.1	492	Yes	-	-	-	-	CCE73854.1	1569	Yes	Yes
4	BMBtpLA3	KX190834.1	37385	ANT40091.1	504	Yes	-	-	-	-	ANT40092.1	1706	Yes	Yes
5	BtCS33	JN191664.1	41992	YP_006488683.1	494	Yes	-	-	-	-	YP_006488684.1	1341	No	Yes
6	BtiUFT6.51-F	MG710484.1	42076	AUO78575.1	494	Yes	-	-	-	-	AUO78576.1	1341	No	Yes
7	Carmel_SA	KY963371.1	40165	ARW58517.1	496	Yes	-	-	-	-	ARW58514.1	1321	No	Yes
8	Cherry	DQ222851.1	36615	ABA46394.1	500	Yes	-	-	-	-	ABA46383.1	1288	No	Yes
9	Deep Purple	MF176161.1	36278	ARW58279.1	495	Yes	-	-	-	-	ARW58280.1	2156	Yes	Yes
10	Fah	DQ150593.1	37974	YP_512323.1	500	Yes	-	-	-	-	YP_512324.1	1280	No	Yes
11	Gamma	NC_007458.1	37253	YP_338197.1	500	Yes	-	-	-	-	YP_338198.1	1288	No	Yes
12	Gamma isolate d'Herelle	DQ289556.1	37373	ABC40466.1	496	Yes	-	-	-	-	ABC40467.1	1331	No	Yes
13	Negev_SA	KY963370.1	40375	ARW58460.1	496	Yes	-	-	-	-	ARW58457.1	1332	No	Yes
14	PBC5	KT070868.1	56332	AKQ08593.1	383	Yes	-	-	-	-	AKQ08594.1	1795	No	No
15	PfEFR-4	KX227757.1	43223	ANT40208.1	494	Yes	-	-	-	-	ANT40159.1	1337	No	Yes
16	PfEFR-5	KX227760.1	43773	YP_009285262.1	494	Yes	-	-	-	-	YP_009285263.1	1337	No	Yes
17	phBC6A51	NC_004820.1	61395	NP_852541.1	172	Yes	-	-	-	-	NP_852542.1	1658	No	Yes
18	phi4B1	KT626446.1	38663	YP_009206319.1	504	Yes	-	-	-	-	YP_009206320.1	1551	Yes	Yes
19	Phi4I1	KT967075.1	41999	ALN97330.1	494	Yes	-	-	-	-	ALN97350.1	1341	No	Yes
20	Phi4J1	KT970645.1	41486	YP_009218149.1	492	Yes	-	-	-	-	YP_009218150.1	1618	Yes	No
21	phiCM3	KF296718.1	38772	YP_009009162.1	494	Yes	-	-	-	-	YP_009009163.1	1302	No	Yes
22	phIS3501	JQ062992.1	44401	YP_007004373.1	494	Yes	-	-	-	-	YP_007004374.1	1341	No	Yes
23	phiS58	KT970646.1	46635	ALO79883.1	492	Yes	-	-	-	-	ALO79942.1	1569	Yes	Yes
24	proCM3	KF296717.1	43278	AGV99416.1	504	Yes	-	-	-	-	AGV99417.1	924	Yes	Yes
25	Tavor_SA	KY963369.1	40397	ARW58399.1	496	Yes	-	-	-	-	ARW58396.1	1332	No	Yes
26	vB_BceS_KLEB30-3S	MT136606.1	37134	QIQ68019.1	504	Yes	-	-	-	-	QIQ68020.1	1860	Yes	Yes
27	vB_BceS-MY192	KT725776.1	44696	ALV83508.1	493	Yes	-	-	-	-	ALV83509.1	1337	No	Yes
28	vB_BtS_B83	MK759918.1	49952	QCQ57801.1	482	Yes	-	-	-	-	QCQ57802.1	1720	Yes	No
29	vB_BtS_BMBtp14	KX190833.1	50740	ANT40030.1	499	Yes	-	-	-	-	ANT40031.1	1394	Yes	No
30	vB_BtS_BMBtp2	NC_019912.1	36932	YP_007236359.1	214	Yes	-	-	-	-	YP_007236360.1	1786	Yes	Yes
31	vB_BtS_BMBtp3	NC_028748.2	51366	YP_009193994.1	494	Yes	-	-	-	-	YP_009193995.2	1570	Yes	Yes
32	Waukesha92	NC_025424.1	45648	YP_009099320.1	492	Yes	-	-	-	-	YP_009099319.1	1098	Yes	Yes

33	WBeta	NC_007734.1	40867	YP_459978.1	496	Yes	-	-	-	-	YP_459979.1	1331	No	Yes
**Organization B**														
34	250	GU229986.1	56505	YP_009219595.1	492	Yes	ADB28367.1	1189	Yes	Yes	YP_009219597.1	461	No	No
35	BMBtp1	KT852578.1	35838	ALJ98009.1	227	No	ALJ98010.1	780	No	Yes	ALJ98011.1	424	No	No
36	vB_BceS-IEBH	EU874396.1	53104	YP_002154388.1	473	Yes	ACH42321.1	1189	Yes	Yes	YP_002154390.1	478	No	No
37	PflS075	KX227759.1	48709	ANT40319.1	492	Yes	ANT40320.1	1194	Yes	Yes	ANT40321.1	497	No	No
38	PfNC7401	KX227758.1	48055	ANT40250.1	492	Yes	ANT40251.1	1194	Yes	Yes	ANT40252.1	439	No	No
39	phBC6A52	NC_004821.1	38472	NP_852600.1	227	No	NP_852601.1*	503	No	No	NP_852603.1	388	No	No
40	vB_BthS_BMBphi	MH458951.1	49277	AXF39895.1	643	Yes	AXF39894.1	518	No	No	AXF39892.1	427	No	No
41	vB_BthS-HD29phi	MN065183.1	32181	QDP43488.1	227	No	QDP43489.1	780	No	Yes	QDP43490.1	393	No	No
42	vB_BtS_BMBtp13	KX190832.1	26471	ANT39954.1	227	No	ANT39955.1	801	No	Yes	ANT39956.1	393	No	No
43	vB_BtS_BMBtp15	KX190835.1	34986	ANT40132.1	227	No	ANT40133.1	780	No	Yes	ANT40134.1	423	No	No
44	vB_BtS_BMBtp16	KT372714.1	34979	ALF01600.1	227	No	ALF01601.1	780	No	Yes	ALF01602.1	422	No	No
**Organization C**														
45	Anath	MG983742.1	52369	-	-	-	AVO23032.1	713	Yes	No	AVO23022.1**	607	No	No
46	Carment17	MG784342.1	41820	-	-	-	AUR81261.1	377	No	No	AUR81258.1	501	No	No
47	PBC1	JQ619704.1	41164	-	-	-	AFE86255	381	No	No	AFE86258.1	429	No	No
48	Wes44	MH598512.1	42248	-	-	-	AXN58349.1	377	No	No	AXN58346.1	503	No	No
**Organization D**														
49	Basilisk	KC595511.2	82008	AGR46592.1	121	No	AGR46593.1	1052	No	No	AGR46595.1	1359	Yes	No
50	PBC4	KT070866.1	80647	AKQ08210.1	121	No	AKQ08211.1	1052	Yes	No	AKQ08213.1	1501	Yes	No
51	pW4	MK288022.1	80919	AZU99066.1	121	No	AZU99067.1	1052	No	No	AZU99069.1	1594	Yes	No
52	v_B-Bak1	MG967616.1	80764	AXY83000.1	121	No	AXY83001	1052	No	No	AXY83003.1	1359	Yes	No
53	v_B-Bak10	MG967618.1	82931	AXY83260.1	121	No	AXY83205	1052	No	No	AXY83204.1	1359	Yes	No
54	v_B-Bak6	MG967617.1	80764	AXY83120.1	121	No	AXY83121.1	1052	No	No	AXY83123.1	1359	Yes	No
**Organization E**														
55	PBC2	KT070867.1	168689	AKQ08520.1	255	No	AKQ08519	899	No	No	AKQ08517.1	983	Yes	No
56	pW2	MK288021.1	160627	AZU98924.1	255	No	AZU98922	887	No	No	AZU98920.1	1439	Yes	No
57	vB_BanS-Tsamsa	KC481682.1	168876	AGI11777.1	255	No	AGI11731	887	No	No	AGI11727.1	1476	Yes	No

***** The IMC is encoded by the gene downstream *tal* (NP_852602.1).

** Displays structural similarities with depolymerases in its C-terminal part.

Table S2: Relevant hits based on the HHpred analysis of TP encoded by phages from the different tail module organizations. One representative phage of each class was selected as model. PDB ID: Protein Data Bank ID number; Prob.: Probability; Id.: Identity; IMC: Intramolecular chaperone, evoDit: Evolved Distal tail protein; CBM: Carbohydrate Binding Module; RBP: Receptor Binding Protein.

Proteins (Size in aa)	Residues (aa)	Hit	PDB ID	Prob. (%)	Id. (%)
**Genetic organization A: phi4B1**					
evoDit (504)	193-445	*Lactobacillus casei* phage J-1 evoDit CBM2, Gp16	5LY8_A	100	18
	1-203<br>444-504	*Bacillus subtilis* SPP1 Dit protein, Gp19.1	2X8K_A	99.8<br>98	20<br>20
	3-207<br>444-504	*Lactococcus lactis* phage TP901-1 Dit protein, orf46	4V96_AV	99.6<br>98	15<br>20
RBP (1551)	4-443	*Staphylococcus aureus* phage 80α Tal, Gp59	6V8I_CE	99.9	13
	1-376	*Listeria monocytogenes* EGD-e prophage tail protein, Gp18	3GS9_A	99.8	12
	683-1042	*Paenibacillus barcinonensis* Xyn10C CBM22-1-CBM22-2	4XUP_A	95.5	11
	682-1042	*Bifidobacterium longum* endo-α-N-acetyl-galactosaminidase	2ZXQ_A	92.8	10
	1404-1527	*Escherichia coli* phage K1F endo-N-acetylneuraminidase IMC	3GW6_A	97.4	15
	1403-1523	*E. coli* phage T5 L-shaped tail fiber with its IMC domain	4UW8_A	97.3	12
**Genetic organization B: vB_BtS_BMBtp13**					
Dit (227)	1-227	*B. subtilis* phage SPP1 Dit protein, Gp19.1	2X8K_C	100	13
	1-227	*S. aureus* phage 80α Dit protein, Gp58	6V8I_BD	100	15
Tal (801)	1-369	*L. monocytogenes* EGD-e prophage tail protein, Gp18	3GS9_A	99.9	16
	1-389	*S. aureus* phage 80α Tal, Gp59	6V8I_CE	99.9	15
	647-775	*E. coli* phage K1F endo-N-acetylneuraminidase IMC	3GW6_A	93.6	16
RBP (393)	1-146	*L. lactis* phage TP901-1 BppU, orf48	4V96_AA	99.7	22
	3-167	*S. aureus* phage 80α lower fiber, Gp62	6V8I_CK	99.6	18
	201-393	*L. monocytogenes* phage PSA RBP C-ter, Gp15	6R5W_B	98.9	18
	313-393	*L. lactis* phage TP901-1 RBP, ORF49	4IOS_A	96.9	14
**Genetic organization B: IEBH**					
evoDit (473)	164-412	*L. casei* phage J-1 evoDit CBM2, Gp16	5LY8_A	99.9	18
	8-157<br>412-472	*B. subtilis* SPP1 Dit protein, Gp19.1	2X8K_C	99.5<br>96.2	23<br>23
	8-157<br>412-473	*L. lactis* phage TP901-1 Dit protein, orf46	4V96_AX	99.4<br>95	13<br>23
Tal (1189)	6-381	*S. aureus* phage 80α Tal, Gp59	6V8I_CE	99.9	13
	3-360	*L. monocytogenes* EGD-e prophage tail protein, Gp18	3GS9_A	99.9	13
	506-688	CBM4 from *Clostridium thermocellum* Cellulase CbhA	3K4Z_A	89.7	10
	933-1168	*E. coli* phage K1F Endo-N-acetylneuraminidase IMC	3GW6_A	95	10
RBP (478)	8-220	*L. lactis* phage TP901-1 BppU, orf48	4V96_AA	99.7	17
	1-157	*S. aureus* phage 80α lower fiber, Gp62	6V8I_CK	99.7	20
	381-463	*L. monocytogenes* phage PSA RBP C-ter, Gp15	6R5W_B	98.1	21
	382-478	*L. lactis* phage TP901-1 RBP, ORF49	4IOS_A	98.9	13

**Genetic organization C: Carmen17**					
YD protein (1057)	338-935	Interleukin-6 receptor subunit beta; Ig-like, *Homo sapiens*	3L5H_A	99.6	14
	538-939	Interferon alpha/beta receptor 1, *Mus musculus*	3WCY_A	99.6	13
	540-934	Fibronectin, *Homo sapiens*	1FNF_A	99.6	14
Tal (377)	24-373	*Shewanella oneidensis* prophage MuSo2 tail protein	3CDD_A	100	10
	29-371	*L. monocytogenes* EGD-e prophage tail protein, Gp18	3GS9_A	99.5	11
	31-371	*S. aureus* phage 80α Tal, Gp59	6V8I_CE	99	12
RBP (501)	6-171	*L. lactis* phage TP901-1 BppU, orf48	4V96_AO	99.6	18
	1-171	*S. aureus* phage 80α Fiber Lower, Gp62	6V8I_FK	99.5	22
	399-501	*L. monocytogenes* phage PSA RBP C-ter, Gp15	6R5W_C	97.4	14
	419-501	*L. lactis* phage TP901-1 RBP, ORF49	4IOS_A	96.6	17
**Genetic organization D: Basilik**					
Dit (121)	1-87	*E. coli* phage T5 Dit protein	6F2M_B	86.8	17
Tal (1052)	10-565	*S. oneidensis* prophage MuSo2 tail protein,	3CDD_A	99.7	10
	14-547	Enterobacteria phage Mu baseplate hub protein Gp44	1WRU_A	99.6	13
	55-545	*S. aureus* phage 80α Tal, Gp59	6V8I_CE	97.7	9
TP (589)	389-589	Drosophila melanogaster, Ig-like domain	6S9F_A	93.6	15
	409-589	Titin; I-set Ig fold, extended poly-Ig; *Oryctolagus cuniculus*	3B43_A	92.6	14
RBP (1359)	541-994<br>290-693	*P. barcinonensis* Xyn10C CBM22-1-CBM22-2	4XUP_D	97.9	11
	539-994	*Streptococcus pneumoniae* endo-alpha-N-acetylgalactosaminidase	5A57_A	96.7	8
TP (593)	98-563	*Homo sapiens*, Interleukin-6 receptor subunit beta; Ig-like	3L5H_A	99.9	11
	107-492	Fibronectin; FN3 domain, *Homo sapiens*	6MFA_A	99.87	12
**Genetic organization E: vB_BanS-Tsamsa**					
Dit (256)	1-255	*B. subtilis* SPP1 Dit protein, Gp19.1	2X8K_A	100	15
	5-255	*S. aureus* phage 80α Dit protein, Gp58	6V8I_CC	100	10
TP (161)	1-161	Cysteine peptidase from *B. cereus* ATCC 10987	3KW0_D	100	27
	1-161	Metalloprotein from *E. coli* O157:H7	2IF6_A	100	25
Tal (887)	14-581	*S. aureus* phage 80α Tal, Gp59	6V8I_AE	100	12
	11-511	*L. monocytogenes* EGD-e prophage tail protein, Gp18	3GS9_A	99.9	16
	27-490	*S. oneidensis* prophage MuSo2 tail protein	3CDD_F	98.6	10
TP (364)	71-364	Titin; I-set Ig fold, extended poly-Ig; *Oryctolagus cuniculus*	2RIK_A	96.4	10
RBP (1476)	910-1370	*P. barcinonensis* Xyn10C CBM22-1-CBM22-2	4XUP_D	97.8	8
	62-223	*L. lactis* phage Tuc2009, BppA orf52	5E7T_B	89.8	15
	60-211	*Cellulomonas fimi* endoglucanase cellulose binding domain	1CX1_A	84.7	15
TP (905)	796-894	*E. coli* phage K1F Endo-N-acetylneuraminidase IMC	3GW6_A	98.3	11
	796-8αα97	*E. coli* phage T5 L-shaped tail fiber with its IMC domain	4UW8_A	98.2	14

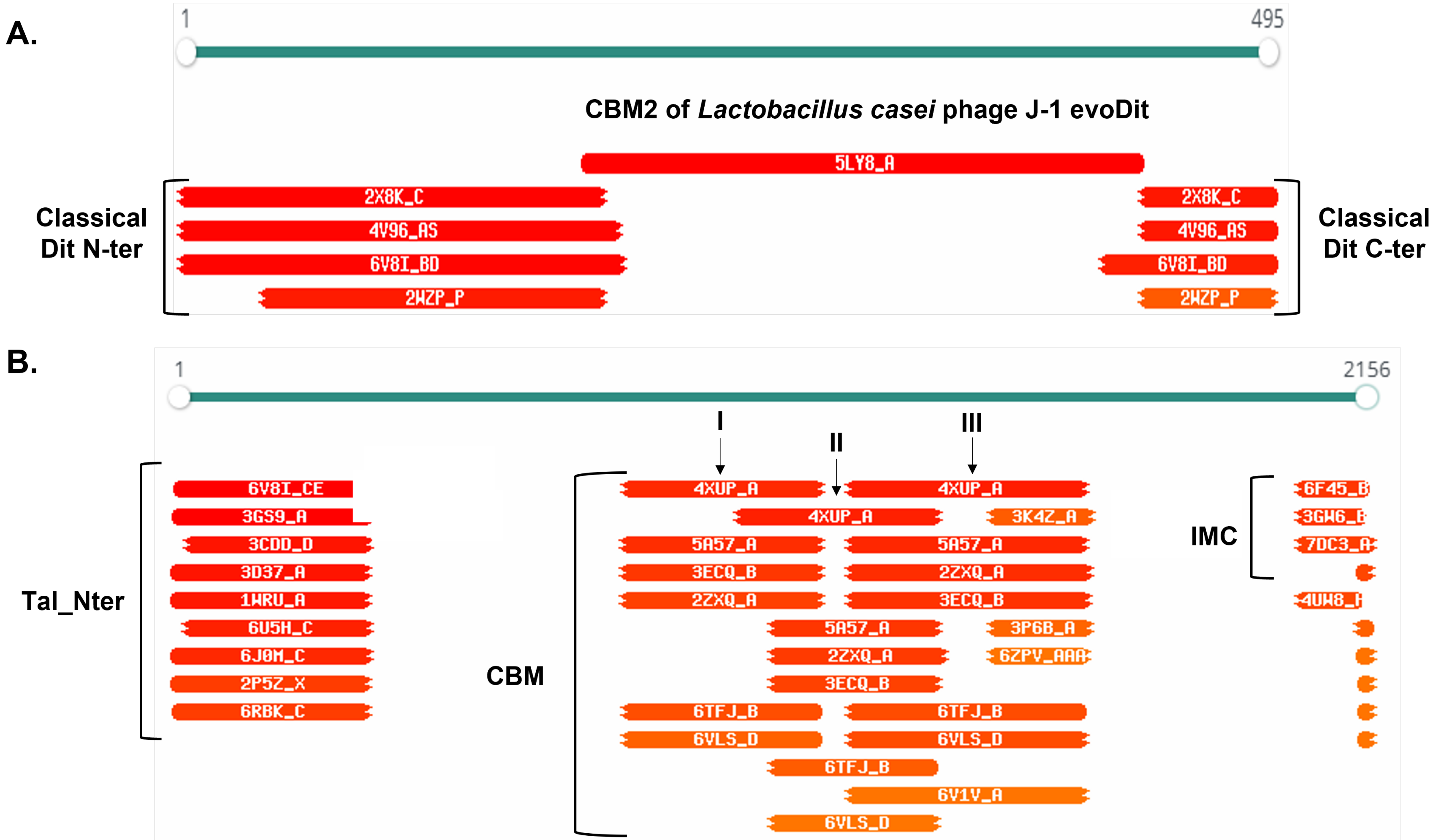

Figure S1: Deep-Purple evoDit-Gp28 and RBP-Gp29 HHpred analysis. A. evoDit-Gp28 shows structural similarities with classical Dit protein in its N- and C- regions while the central part is occupied by a region structurally similar to the CBM2 found in *L. casei* phage J-1 evoDit. **B.** RBP-Gp29 has structural homologies with the N-terminal part of various Tal in its N-terminal end, a central region in which different folds correspond to different CBM and a C-terminal part displaying IMC hits. I, II and III indicate similar CBM found in tandem repeats. ID numbers corresponding to the different protein structures in the Protein Data Bank are indicated for each hit.

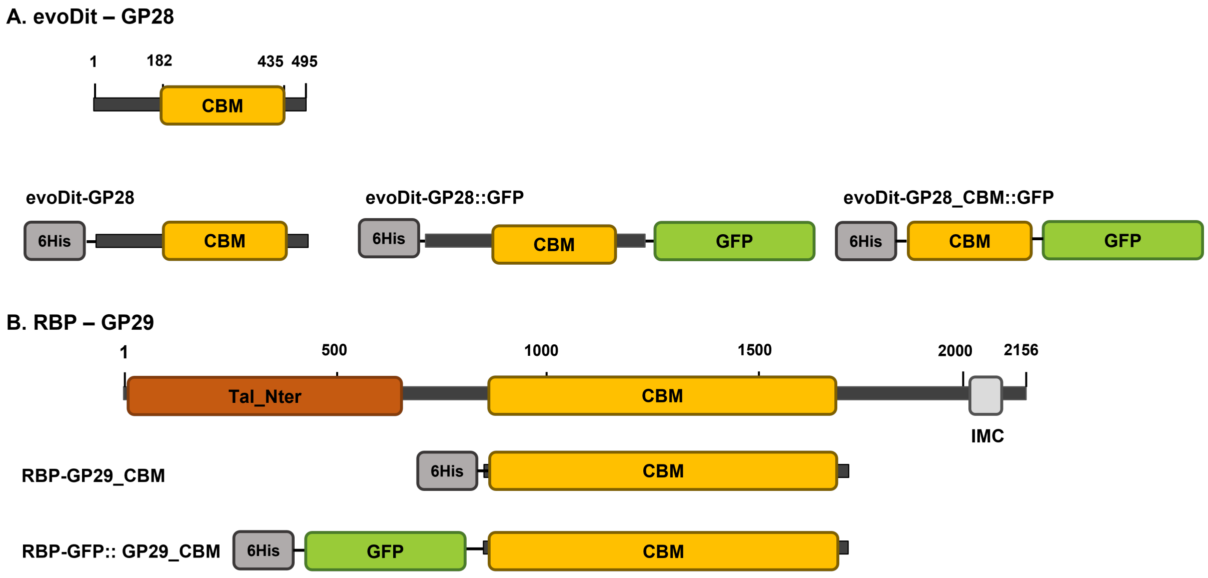

Figure S2: Deep-Purple evoDit-Gp28 and RBP-Gp29 and derived fusion proteins. A. Domain organization of the evoDit-Gp28 and corresponding protein constructions. **B.** Domain organization of the RBP-Gp29 and corresponding protein constructions. The numbers refer to the residue coordinates. CBM: Carbohydrate Binding Module; 6His: Six histidine Tag; GFP: Green Fluorescent Protein; Tal_Nter, N-terminal domain of Tail Lysin; IMC: Intramolecular chaperone.

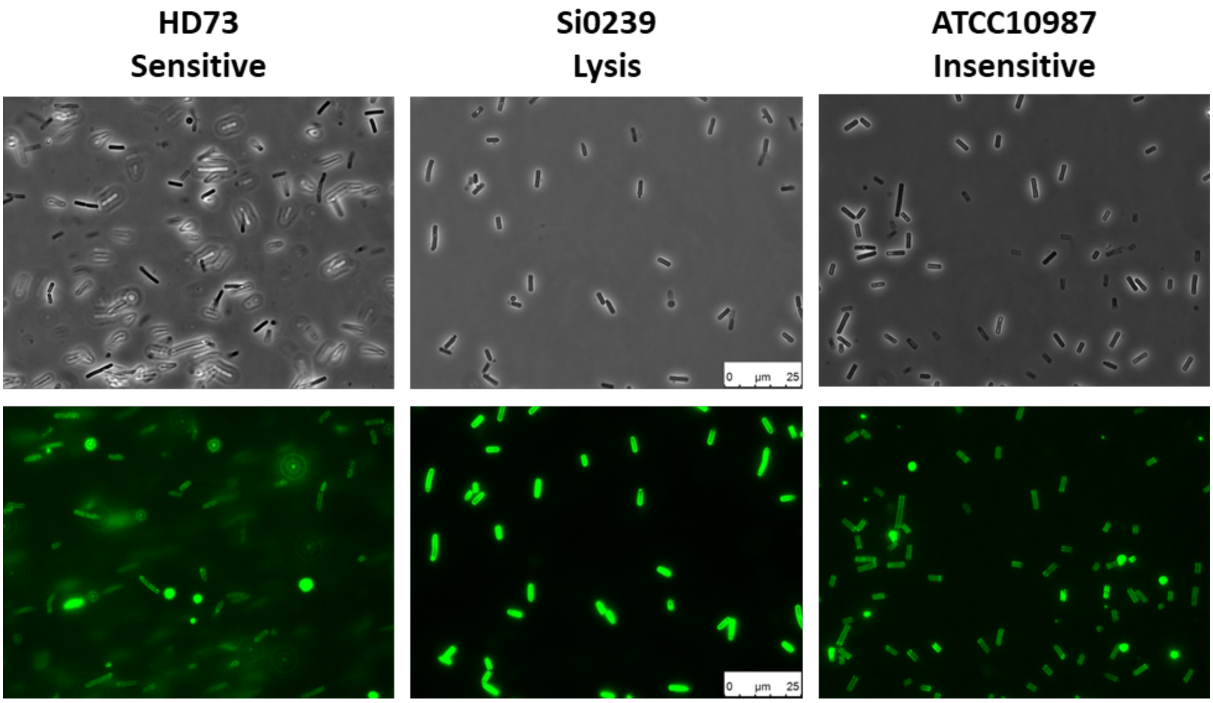

Figure S3: Cell wall binding assay of evoDit-Gp28_CBM to *B. cereus* cells. The central CBM of Gp28 was fused to a GFP tag to assess its role in the adsorption of Gp28 to *B. cereus*. Strains sensitive and insensitive to the phage, as well as strains affected by lysis from without, were tested in a cell wall decoration assay (Table 2). The upper row shows bright field microscopy images and the lower row displays the corresponding fluorescent images. The scale bar is identical for all pictures.
